# Cerebral Meningitis (Intercurrent)

**Published:** 1875-07

**Authors:** Walter Hay

**Affiliations:** No. 163 State St.; Chicago


A MONTHLY RECORD OF

*Medicine, Surgery and the Collateral Sciences.*

EDITED BY J. ADAMS ALLEN, M.D., LL.D.: AND WALTER HAY, M.D.

VOL. XXXII. — JULY, 1875. — NO. 7.

### Original Communications.

ARTICLE I.

### CEREBRAL MENINGITIS (INTERCURRENT).

BY WALTER HAY, M.D., CHICAGO.

A. McM., æt. 10 years; Jan. 10, 1875, the third day of an attack of measles, had moderate eruption over face, neck, chest and arms, slight febrile reaction, the usual coryza and catarrh. Was ordered the following: ℞. Liq. Ammon. Acet., ʒj, every two hours; and ℞. Syr. Scillar, gtts. xxv; Spts. Ætheris Nitrosi, gtts. xv. M. Every alternate two hours.

January 11 and 12, was progressing satisfactorily, but as she did not sleep well last night, was ordered to take Chloral Hydrat. grs. v, in water, at bedtime; the treatment to be continued otherwise.

Jan. 13. Slept well last night, and at 10 o'clock was less feverish; more comfortable generally. At 12 M. was seized suddenly with intense headache, delirium, vomiting and unilateral convulsions, involving the face and both extremities of the right side, the right eye being drawn upward and outward (3d, 4th and 6th nerve implicated), (such was the report of the Sister of Charity in charge). At 3 P. M. she was dull and stupid, indicating by gestures pain in the head, and was roused to attention with great difficulty; the eyelids were half closed, the pupils dilated and responding very slowly to strong sunlight, the irides of both eyes reduced to about one-half a line in width, the vessels of the sclerotic injected; the face was deeply flushed, the tongue covered with thick white fur, and protruded with great difficulty; the bowels were constipated, the urine scanty and loaded with amorphous urates. Pulse, 120; respirations, 33; temperature, 101.8° Fah. ℞. Hydrarg Sub-Mur., gr. j; Quiniæ Sulph., gr. j. M. Every two hours. Also, ℞. Fl. Ext. Secale Cornut., gtts. x. Every alternate two hours. Ice to head, and the discontinuance of all other treatment.

Jan. 14, 10 A. M. Had rested tolerably during the night; the bowels and kidneys had acted copiously at 6 A. M. Her countenance is bright; she smiles when spoken to; the pupils are contracted to one-half their former diameter, although still somewhat dilated; the vessels of the sclerotic no longer visible; has no headache; pulse, 80; respirations, 20; temperature, 98°; has had no repetition of the convulsions. ℞. Fl. Ext. Secale Cornut., gtts. x. Every four hours.

Jan. 15. Convalescent. Discontinue treatment.

This case is reported as an unusual case of incipient cerebral meningitis supervening in the course of a fully developed exanthem, without retrocession of the eruption. Sidney Ringer (Reynolds' System of Medicine, London edition, 1870, vol. 1, page 196; article Measles, Complications and Sequelæ,) says: "Convulsions, not uncommon in children at the very commencement of the attack, are usually without danger. They may, however, be repeated and terminate fatally. Occurring, by no means commonly, at a later period of the disease, they are then usually repeated, and accompanied with some severe internal inflammation, mostly of the lungs. At this period they are generally fatal."

The case in point could not be included in the first category, although it might be in the second, allowance being made for the difference in the location of the accompanying inflammation, i.e., in the cerebral membranes, instead of the lungs. Ringer, however, in the passage quoted above, evidently indicates "convulsion" as the essentially important complication, whereas in the case which I have reported above, convulsion was but an epiphenomenon of a meningitis already established, and indicated by the headache, delirium and vomiting, which preceded the convulsion.

That this was the true pathological relation was, I think, demonstrated by the effect of remedies, more especially of the ergot, which I am persuaded would not have prevented the repetition of the convulsion, as it did, had that been other than a secondary lesion.

Dr. Frederick T. Roberts (Handbook of Practical Medicine, Am. edition, 1874, page 188; article Measles, Varieties; 4. Graviores—malignant, black, or hæmorrhagic) says: "There is great depression and prostration, with a very weak, frequent and irregular pulse, cold extremities, a dry and brown tongue, and sordes on the teeth and lips; twitchings, picking at the bed-clothes, *convulsions*, *delirium of a low* and muttering character, or stupor, are generally present very early." A description which in no way applies to the case reported.

Trousseau (Lectures on Clinical Medicine, third (Am.) edition, 1869, page 218, article Measles), speaks of convulsions occurring at the onset of the fever, of measles, and of many other affections, being simply a continuation or intensification of the rigor which usually initiates these maladies, remarking, very justly, that rigor is "really a convulsion," the one clonic, the other tonic. And again, the same author, on page 235, refers to a repetition of these convulsions "in the last stage of the disease," "preceded or followed by cerebral disturbance characterized by stupor," "which generally carry off the patient;" and to "the nervous complications of the last stage of measles which originate in formidable chest affection." The author, however, nowhere refers to any condition at all comparable to the case reported herein.

Flint, (Principles and Practice of Medicine, 4th ed., 1873, page 993, article Measles) says: "Convulsions sometimes occur in this stage (of invasion). In general, they are not indicative of danger." Upon the subject of cerebral complications, otherwise, he is silent.

Niemeyer (Text-Book of Practical Medicine, Am. revised ed., 1874, page 527, article Measles) refers incidentally to "pain in the head, . . . vomiting, disturbed sleep, and in excitable children, delirium," as occurring "also during simple catarrh from catching cold;" and again, on page 531, referring to "asthenic septic measles," he says, "the patient may die of the increasing prostration which is occasionally interrupted by *eclamptic spasms* even before the appearance of the eruption."

Thomas (Ziemssen's Cyclopædia of the Practice of Medicine, first ed. (Am.), 1875, page 97, article Measles), says: "Conspicuous among the affections of the nervous system are: Meningitis (Spiess, Voit, Meyer-Hoffmeister, Kellner, Constant, Loschner, Thore, Bufalini, Krug); . . . . . tonic spasms (especially in the flexors of the extremities, rolling spasms of the head, two days after the eruption, accompanied by its disappearance: Pinkham); convulsions and eclampsia, usually with similar effect on the exanthem at different periods of the same."

From these citations it seems clear that, the cerebral symptoms, complicating the attack of measles in this child, are unfamiliar to the profession in England, France and America, since the most recent standard authorities are silent upon the subject—for the pathological conditions indicated by Ringer, Roberts, Trousseau and Flint vary essentially from that of the subject of this report, nor do those described by Niemeyer correspond much more closely. The latter author is probably the best known among Germans to American students, while the writings of those quoted by Thomas (Ziemssen's Cyclop.) are doubtless, to the vast majority of English speaking and reading physicians, sealed books.

Having met with no similar case, amongst some hundreds observed during more than twenty years of practice, I was induced to examine the literature of the subject as far as it was accessible to me, to determine how far my own experience might be sustained or refuted by other observers, with the results above recorded.

But, that this was actually an intercurrent meningitis, may be disputed by some. On this head, the definition of Trousseau (op. cit., vol. 1, page 527) is sufficiently explicit: "I need not remind you that the invasion of ordinary meningitis is generally announced by vomiting, pain in the head, which is sometimes fearful, by constipation, and in children by convulsions;" than which a more accurate epitome of the phenomena of this case could not be drawn.

No. 163 State St.

